# The Surgeon's Role in the Opioid Crisis: A Narrative Review and Call to Action

**DOI:** 10.3389/fsurg.2020.00004

**Published:** 2020-02-18

**Authors:** Cade Shadbolt, J. Haxby Abbott, Ximena Camacho, Philip Clarke, L. Stefan Lohmander, Tim Spelman, Eric C. Sun, Jonas B. Thorlund, Yuting Zhang, Michelle M. Dowsey, Peter F. M. Choong

**Affiliations:** ^1^Department of Surgery, St. Vincent's Hospital, The University of Melbourne, Melbourne, VIC, Australia; ^2^Centre for Musculoskeletal Outcomes Research, Department of Surgical Sciences, Dunedin School of Medicine, University of Otago, Dunedin, New Zealand; ^3^Melbourne School of Population and Global Health, The University of Melbourne, Carlton, VIC, Australia; ^4^Centre for Health Policy, Melbourne School of Population and Global Health, The University of Melbourne, Carlton, VIC, Australia; ^5^Health Economics Research Centre, Nuffield Department of Population Health, University of Oxford, Oxford, United Kingdom; ^6^Department of Clinical Sciences Lund, Orthopaedics, Lund University, Lund, Sweden; ^7^Department of Clinical Neuroscience, Karolinska Institute, Stockholm, Sweden; ^8^Rigshospitalet, Copenhagen, Denmark; ^9^Department of Anaesthesiology, Perioperative and Pain Medicine and Department of Health Research and Policy, Stanford University, Stanford, CA, United States; ^10^Department of Sports Science and Clinical Biomechanics, University of Southern Denmark, Odense, Denmark; ^11^Research Unit for General Practice, Department of Public Health, University of Southern Denmark, Odense, Denmark; ^12^Melbourne Institute of Applied Economic and Social Research, Faculty of Business and Economics, University of Melbourne, Carlton, VIC, Australia; ^13^Department of Orthopaedics, St. Vincent's Hospital, Melbourne, VIC, Australia

**Keywords:** opioids, surgery, prescribing practices, risk factors, postoperative opioid use

## Abstract

Over the past two decades, there has been a sharp rise in the use of prescription opioids. In several countries, most notably the United States, opioid-related harm has been deemed a public health crisis. As surgeons are among the most prolific prescribers of opioids, growing attention is now being paid to the role that opioids play in surgical care. While opioids may sometimes be necessary to provide patients with adequate relief from acute pain after major surgery, the impact of opioids on the quality and safety of surgical care calls for greater scrutiny. This narrative review summarizes the available evidence on rates of persistent postsurgical opioid use and highlights the need to target known risk factors for persistent postoperative use before patients present for surgery. We draw attention to the mounting evidence that preoperative opioid exposure places patients at risk of persistent postoperative use, while also contributing to an increased risk of several other adverse clinical outcomes. By discussing the prevalence of excess opioid prescribing following surgery and highlighting significant variations in prescribing practices between countries, we note that there is a pressing need to optimize postoperative prescribing practices. Guided by the available evidence, we call for specific actions to be taken to address important research gaps and alleviate the harms associated with opioid use among surgical patients.

## Introduction

Over the past two decades, rates of opioid use have risen sharply around the world. Between 2001–03 and 2011–13, global opioid use doubled to more than 7.3 billion daily doses per year (1). The effects of this worrying trend have been most pronounced in the United States, where opioid misuse has become a national health concern (1, 2). Similar trends have emerged in Canada (3), parts of Central and Western Europe (1), and Australia (4, 5). Even in Norway and Sweden, where the overall prevalence of prescription opioid use has been comparatively stable over the last decade, there has been an increase in both the availability of the strong opioid oxycodone and oxycodone related deaths (6, 7).

The human and economic costs of opioid misuse are substantial. With more than 43,000 Americans dying from opioid overdoses in 2017 alone, opioids now kill more people than motor vehicle accidents in the US (8). Patients who are prescribed long-acting opioids face an increased risk of all-cause mortality relative to those prescribed other common pain medications (9), and upwards of 40% of suicide and overdose deaths in the US have a documented link to opioids (10). The total economic burden of opioids in the US alone was recently estimated to be $504 billion per year (11). This headline figure included $431.7 billion in costs associated with fatal opioid-involved overdoses, and $72.3 billion in costs associated with non-fatal abuse and dependence due to increased healthcare, substance abuse treatment, and criminal justice spending, as well as reductions in productive employment (11, 12).

A growing amount of attention is rightly being paid to the widespread practice of prescribing opioids for postsurgical analgesia. In the US, surgeons wrote 28.3 million opioid prescriptions in 2012, which accounted for 9.8% of all opioid prescriptions written that year (13). In Australia, surgeons are responsible for 6.6% of patients being initiated onto opioid therapy each year (4). While opioids may be necessary to provide patients with adequate relief from acute pain after major surgery, their role at and beyond discharge calls for greater scrutiny. To make informed changes to the practice of postsurgical opioid prescribing, it is vital that we first understand the need for these changes, particularly in the setting of the risks associated with preoperative opioid use among surgical patients, the rate of persistent postsurgical opioid use, and risk factors associated with persistent use (see Box 1). We must also be aware of how often excess opioids remain unused after surgery, and of what guidelines are currently available for postsurgical prescribing. This narrative review aims to provide an overview of the growing literature on these topics. Guided by the current evidence, we call for actions to be taken to address important research gaps and alleviate the harms associated with opioid use following surgery.

Box 1 **Defining the problems.**

**Opioid misuse:** Opioid use that diverges from the prescribed pattern of use (14). This broader concept will include instances of opioid abuse and dependence.

**Opioid abuse:** Intentional opioid misuse that is for a non-medical purpose. This includes misuse that aims to elicit feelings of euphoria or alter one's state of consciousness (14).

**Opioid dependence:** A cluster of symptoms that may develop following repeated opioid use. Typical symptoms include difficulty controlling use, cravings, increased tolerance, physical withdrawal, and persistent use despite harmful consequences (15).

**Persistent postoperative use:** Opioid use that continues beyond the point at which acute postsurgical pain is expected to have resolved. While exact definitions vary, a recent consensus statement defines persistent use among opioid-naïve patients as the use of opioids for 60 days between postoperative days 90–365 (16).

**Excess opioid prescribing:** Prescribing a quantity of opioids that is more than the quantity that is ultimately consumed by the patient. Importantly, it is possible to inappropriately prescribe opioids (or prescribe a greater dosage than is necessary) even when the patient consumes their entire prescribed dose. For the purposes of this paper, this concept refers to prescribing that results in excess unused opioids rather than the medically unnecessary or suboptimal prescribing of opioids.

## Persistent Use of Opioids After Surgery

Against the backdrop of opioid crises around the world, it is concerning that many patients who are prescribed opioids to alleviate acute postoperative pain progress to longer-term use. Initiating this transition to persistent postoperative use is likely one of the primary ways in which surgeons have contributed to opioid-related harm in the community. This is made all the more worrying by the fact that recent guidelines have emphasized that there is no strong evidence to support the effectiveness of long-term opioid therapy—but extensive evidence that long-term use is associated with substantial risks (17, 18). These include increased risk of opioid abuse or dependence, fatal and non-fatal overdose, endocrinological harms, cardiovascular events, and road trauma (17).

Despite this, there does not appear to be a widely accepted definition of persistent postoperative opioid use (16). Some definitions that have appeared in the literature include: continued use for more than 90 days after discharge (19), filling 10 or more prescriptions in the year after surgery (20), and either 90 days of continuous use or 120 days of non-continuous use after surgery (21). In an effort to standardize the definition of persistent postoperative use, the American Society for Enhanced Recovery recently issued a consensus statement (16). In this statement, persistent postoperative opioid use was defined differently for opioid-naïve and non-naïve patients. In opioid-naïve patients, persistent use was defined as using opioids for at least 60 days in the 90–365 days postoperative period. In non-naïve patients, persistent postoperative use was defined as an increase in opioid use in the 90–365 days postoperative period when compared to their use in the 90 days before surgery. The uptake of some form of standard measure would undoubtedly make future research among different populations more readily comparable and better placed to inform changes to health policy or prescribing practices.

Two recent systematic reviews have reported rates of persistent opioid use following surgery (16, 22). Mohamadi et al. (22) reported an overall rate of prolonged use of 4.3% (95% CI 2.3–8.2%) among 1.72 million surgical and trauma patients examined in 14 large population-based studies. Kent et al. (16) reviewed 47 studies that evaluated patients who had undergone procedures from five surgical subgroups (Table 1). From the moderate quality studies assessed, incidence of persistent postoperative use ranged from 0.6 to 26% for opioid-naïve patients and from 35 to 77% for non-naïve patients (16). While these reviews highlight the considerable number of studies that have quantified rates of persistent use, it is worth noting that there has been little qualitative research that specifically examines patients' experiences of persistent postoperative opioid use or clinicians' perceptions of the need for opioids after discharge from surgery. Such in-depth research could provide richer insight into the factors that contribute to patients' long-term reliance on opioids after surgery.

**Table 1:** Incidence of persistent postoperative opioid use in moderate quality studies reviewed by Kent et al. (16).

**Surgery**	**Opioid naïve sample (%)**	**Opioid non-naïve sample (%)**	**Mixed sample (%)**
Total hip or knee replacement	0.6–4	35–68	5.5–32
Abdominopelvic	0.12–6	59–77	0.36–14
Spine	26	59	18–59
Mastectomy	10–11	–	–
Thoracic	14	–	–

Both reviews indicated that a non-trivial proportion of surgical patients are at risk of becoming persistent opioid users. Importantly, these reviews also highlighted several patient-related risk factors for postoperative use, including opioid use prior to surgery, benzodiazepine use, pain catastrophizing, depression, smoking, and preoperative pain conditions (16, 22). In addition to this, while both major and minor surgeries place patients at risk of developing persistent opioid use, Mohamadi et al. (22) reported that more invasive procedures were associated with greater odds persistent postoperative use. Each of these risk factors can be understood as a potential target for interventions aimed at reducing rates of persistent postoperative use. To date, the possibility of reducing persistent postoperative use by addressing known risk factors in the preoperative period has been largely overlooked. Research that aims to fill this gap has the potential to substantially reduce rates of prolonged reliance on opioids after surgery.

## Risks Associated With Opioid Use Prior to Surgery

The most prominent risk factor for persistent postoperative use reported in both systematic reviews mentioned above is the use of opioids before surgery. Rates of persistent postoperative use reported by Kent et al. (16) were more than 10 times higher in non-naïve hip and knee replacement, and abdominopelvic patients when compared to opioid naïve patients. Mohamadi et al. (22) reported that preoperative use was associated with roughly 11 times greater odds of long-term postoperative use (OR = 11.04 [95% CI 9.39–12.97]). Preoperative opioid use has also been linked to poorer outcomes across most major surgical specialties (23–30). These adverse outcomes have been widely documented using measures such as length of stay (27, 30), readmission rate (23, 27, 28), postoperative cost of care (24, 25, 31), and complication rate (25). Among orthopedic patient populations, who often have high rates of use prior to surgery (32), preoperative opioid abuse and dependence is associated with significant increases in morbidity and mortality after surgery (33). Among patients undergoing total joint replacement, preoperative opioid use is associated with less postoperative improvement in patient reported pain and function (34). Furthermore, recent studies have found that preoperative opioid users who undergo joint replacement are significantly more likely to suffer a periprosthetic joint infection (35) or require surgical revision (26, 36). The mechanisms through which preoperative opioid use contributes to complications such as joint infection and early revision are almost certainly multifactorial. However, higher rates of postoperative opioid consumption among preoperative users and the immunosuppressive nature of some opioids have been suggested as key contributory factors (35, 36). Given the many ways in which preoperative opioid use may negatively impact clinical outcomes, it is likely that paying careful attention to this driver could contribute to improving the safety and quality of surgical care.

In the most general terms, there are three ways in which preoperative opioid use may be altered prior to surgery. The first, and most wide ranging, is to target the inappropriate prescribing of opioids among all patient populations. Lowering the overall prevalence of opioid use among the population as a whole would potentially be the most viable long-term strategy for reducing the rate of opioid use among patients presenting for surgery. Furthermore, there are particular surgical populations in which the reduction of inappropriate opioid prescribing is likely to drastically reduce the risks associated with preoperative opioid use. For instance, the rate of preoperative opioid use in total knee replacement patients has been reported at roughly 55% (37). This is despite guidelines strongly recommending against the use of opioids in light of strong evidence that opioids offer limited or no relevant benefit in the treatment of osteoarthritis (38). In fact, several randomized controlled trials have shown opioids to be no more effective in treating osteoarthritis-related pain than non-opioid analgesics such as acetaminophen or non-steroidal anti-inflammatories (39, 40). This suggests that increased uptake of evidence-based non-surgical treatment of knee and hip osteoarthritis could potentially eliminate preoperative opioid use among this specific and sizable population (38). Although such a shift may not impact all surgical populations to the same extent, it is imperative that measures be taken to limit inappropriate opioid prescribing whenever possible.

The second way that surgeons may attempt to reduce rates of preoperative opioid use is through timing elective surgeries prior to opioid initiation. In many instances this strategy will be infeasible. However, earlier progression to surgery may be possible for patients with degenerative conditions such as end-stage osteoarthritis where opioids may be inappropriately prescribed in an effort to delay or avoid inevitable surgery, despite strong recommendations against their use (38, 41). Such a pragmatic strategy concedes that inappropriate prescribing is not something that can be controlled entirely by optimal surgical care, and views delayed surgery as increasing the likelihood that patients will be prescribed opioids by another provider. A small amount of health economic research has shown that avoiding opioids through earlier surgery is a potentially cost-effective means of treating knee osteoarthritis (42) and the findings of this research stands to be tested in a variety of settings with this and other procedures. To date, there have been no studies that assess the feasibility or efficacy of such an approach among any surgical populations, targeting this as an important area for future research.

The third broad way that preoperative opioid use may be altered is through tapering or weaning patients from these medications prior to surgery (43). Unlike the first two strategies, this approach aims to respond to patients who have already been exposed to opioids, rather than preventing preoperative opioid initiation. Once again, there is little evidence about the feasibility or effectiveness of preoperative opioid weaning. In the only published study investigating links between preoperative weaning and surgical outcomes, patients who successfully weaned their dose by at least 50% before total joint replacement achieved improved clinical outcomes compared to patients who did not taper their use (44). Importantly, this study did not assess the impact of preoperative weaning on postoperative opioid use. Furthermore, the retrospective design of this study means it is possible that the observed differences are explained by variances in patient characteristics (e.g., tendency to comply with care) among those patients who successfully tapered their use compared with those who did not. Preoperative weaning interventions may also be infeasible because available evidence has not yet clarified how to best support patients to taper their opioid use (45). What's more, opioid tapering is not necessarily a risk-free endeavor, as there are concerns about it resulting in patients being at an increased risk of suicidal thoughts and behaviors (46), or transitioning to heroin use (10, 47). There is clearly a need for further research into the safety, efficacy, and feasibility of preoperative opioid tapering, given that such an intervention may both reduce rates of persistent opioid use and improve surgical outcomes more generally.

## What Do We Know About Post-Operative Prescribing?

### Variations in Postoperative Opioid Prescribing

Opioids are one of the medicines most commonly prescribed by surgeons. In 2012, more than a third of prescriptions written by American surgeons were for opioids (13). Despite such a large portion of surgical prescriptions being for this one class of drug, there is little uniformity in how they are prescribed (48–50). Variability in prescribing practices has been documented in many different domains. In a recent study of pediatric umbilical hernia patients, rates of opioid prescribing varied significantly between census regions, with a rate of 42% in the Northeastern and 59% in the Southern United States (50). It is important to note that such disparities do not only exist on a regional level. In a study of 642 patients undergoing five different common surgical procedures at a single academic medical center, there was wide variation documented between different operations (49). There was also wide variation in prescriptions for patients undergoing the same procedure even when the procedure was conducted by the same provider (49). There is little direct research examining whether similar variation exists in surgical prescribing practices outside of the United States. However, given that significant regional variations in general opioid prescribing has been documented in several other countries (51, 52), it is reasonable to also expect similar variability in postsurgical prescribing.

In light of significant variations in the prevalence of prescription opioid use between countries (1), it is likely that surgical prescribing practices differ substantially between countries. Despite this, there has been a very limited amount of research that directly compares surgical prescribing patterns between countries, and until recently sample sizes in these studies have been small. In one study comparing opioid prescribing practices after operative treatment of hip and ankle fractures in 190 American and 116 Dutch patients, 82% of American patients and 6% of Dutch patients with ankle fractures were prescribed post-discharge opioids (53). Among hip fracture patients, 77% of the American and none of the Dutch patients were prescribed opioids after discharge. Another study, which compared opioid prescriptions for 820 patients undergoing head and neck surgeries in the US and Hong Kong, found that almost none of the patients in Hong Kong were prescribed opioids pre- or post-operatively (54). Recently, Ladha et al. (55) published the first large-scale cohort study comparing postoperative opioid prescribing between countries. By comparing opioid prescriptions following four minor surgical procedures in the US (129,379 patients), Canada (84,653 patients), and Sweden (9,802 patients), this study found opioid prescriptions in the US and Canada were filled at a 7-times greater rate than in Sweden. These findings highlight this as an important domain for future research. Such research could go beyond evaluating differences in prescribing after surgical procedures, to explore differences in patient satisfaction with pain control, surgical outcomes, and clinicians' attitudes toward postoperative opioid prescribing. Observing variations in opioid prescribing both regionally and internationally is a vital preliminary means of assessing the feasibility of offering high quality surgical care while minimizing the pervasiveness of post-discharge opioid analgesia.

### Unused Opioids After Surgery

Several studies have documented the over-supply of opioids after surgery. A systematic review that identified six studies reported that between 42 and 71% of prescribed opioid pills went unused (56). Another review, which identified 11 patient survey studies, found that between 40 and 94% of prescribed pills went unused, with one outlier reporting roughly 10% unused (57). Recently, Sabatino et al. (58) reported on a telephone survey of 198 patients who had undergone a total hip replacement and 146 had undergone a total knee replacement. Approximately 29% of prescribed pills were unused by hip replacement patients and 18% were unused by knee replacement patients. While these findings fall outside the ranges reported by the earlier reviews, this deviation was likely influenced by the fact that none of the studies included in the earlier reviews examined excess prescribing following total hip and knee replacement. To determine how the type of procedure impacts rates of excess opioid prescribing, and to properly inform surgeons' prescribing practices, further research in this domain is clearly warranted. To this end, Thiels et al. (59) recently published a study evaluating rates of excess prescribing following 25 elective procedures, which found that overall 61% of prescribed opioids went unused. Despite the importance of this work, our understanding of excess prescribing following many procedures is still limited by relatively small samples. For instance, our current understanding of the rate of excess prescribing following hip replacement is drawn from two studies reporting on 348 total patients treated in four American hosptials. The narrow scope of this evidence, along with the fact that none of the research cited here has evaluated patient populations outside of North America, highlights an opportunity for novel research into the oversupply of opioids after surgery in other regions.

Reducing excess prescribing is likely to help combat opioid-related harm in at least two ways. First, reducing the size of postoperative opioid prescriptions has been associated with a reduction in the amount of opioids consumed by patients (60). Although the precise mechanism for this reduction in opioid consumption is not known, it is possible that it is a result of patients anchoring and adjusting their expectations to the smaller total prescription size (60). Second, minimizing excess prescribing may reduce the amount of opioids that are diverted to non-medical use. The reviews mentioned above reported that between 4 and 59% of patients planned proper disposal of their excess opioids (56, 57), which leaves a substanital portion of excess opioids available for diversion. Diversion resulting from large opioid prescriptions can have severe consequences. A recent study by Khan and colleagues reported that family members of patients who have been prescribed opioids face between 2.7 (OR, 2.71 95% CI, 2.42–3.03) and 15.1 (OR, 15.08 95% CI, 8.66–26.27) times greater odds of overdosing, with stronger prescriptions being associated with greater odds of overdose (61). Combatting both excess prescribing and poor disposal practices is likely the best way to reduce surgeons' contributions to the harms associated with opioid diversion.

## Summing Up: A Call for Further Research and Action

The available evidence calls for action in two domains (see Box 2).

Box 2 **Evidence gaps.**

**Targeting risk factors prior to surgery** 

- Is targeting known risk factors prior to surgery a viable or effective means of reducing the rate of persistent use following surgery?
- Can opioid-sparing multimodal analgesia after discharge reduce the prevalence of persistent postoperative opioid use or the risk of opioid-related harms following surgery?
- Can risks associated with preoperative opioid use be reduced through preoperative opioid tapering?
- Is earlier progression to elective surgery a viable and effective means of reducing patient's risk of being exposed to opioids prior to surgery?
- What are the patterns and sources of preoperative opioid use?
- Precisely what are the risks associated with preoperative opioid use?

**Optimizing postoperative opioid prescribing practices** 

- What postoperative interventions effectively reduce rates of persistent postoperative opioid use?
- Do available guidelines for postoperative opioid prescribing reduce the prevalence of opioid-related harms following surgery? Do these guidelines reduce rates of persistent postoperative opioid use?
- Do rates of postoperative opioid prescribing differ between countries?
- Do rates of persistent postoperative opioid consumption vary between countries?
- To what extent do opioids prescribed after surgery remain unused by patients outside of North America?

First, surgical care must become more responsive to risk factors associated with persistent postoperative opioid use. The literature has identified several important risk factors; however, to date, there have been no rigorous evaluations of interventions that specifically aim to target these risk factors. Combatting the most significant known risk factor for persistent postoperative use—opioid use prior to surgery—will require action on many fronts. First and foremost, it is essential that inappropriate opioid prescribing is minimized among surgical populations that are at high risk of being prescribed opioids prior to surgery. Despite limited evidence of any benefit and strong evidence of dose-dependent risks (62), vast numbers of patients are still being prescribed opioids for chronic non-cancer pain such as that caused by end-stage osteoarthritis. This poses the question: how do we ensure that what we already know is put into practice? The design and implementation of interventions in this domain ought to be informed by multidisciplinary and qualitative research exploring why clinicians across all major specialties prescribe opioids inappropriately, and why patients perceive the need for opioids prior to surgery. Evaluating the potential efficacy of interventions that aim to reduce inappropriate opioid prescribing at such a general level will require high quality retrospective data, as sufficiently powered clinical trials are likely to be infeasible. In addition to this, surgeons may wish to be mindful of the timing of elective surgeries to minimize the risk that patients will be initiated onto opioids by another provider. This may include such practices as closely monitoring and managing surgical waiting lists to ensure timely surgery. To date, no studies have assessed the impact of reducing waiting times to surgery and its impact on post-operative opioid use, or how this strategy of reducing waiting times may reduce preoperative opioid intake. Finally, as many patients presenting for surgery have already been initiated onto opioids, there is a pressing need for rigorous clinical trials to evaluate the safety, efficacy, and feasibility of preoperative opioid tapering, given that such an intervention may reduce rates of persistent postoperative opioid use while also improving surgical outcomes more generally.

Second, post-discharge prescribing practices need to be optimized to limit patients' risk of persistent postoperative use. The prevalence of excess prescribing and wide variations in prescribing practices indicate that there is substantial scope to improve current post-discharge prescribing practice (48, 50). One approach to optimizing prescribing practices is ensuring that patients are discharged home with carefully constructed multimodal analgesia regimens (e.g., NSAID, Acetaminophen, Gabapentin), to minimize reliance upon opioids following discharge. Multimodal analgesia has consistently been shown to reduce in-hospital opioid consumption (63). However, to date, there is a little research examining multimodal analgesia's impact on persistent postoperative opioid use or its long-term efficacy following surgery (64). In addition to this, the widely publicized successes of the CDC's *Guideline for Prescribing Opioids for Chronic Pain* indicate that guidelines can have a significant positive impact on opioid prescribing practices (65). A limited number of guidelines have been proposed for postsurgical opioid prescribing (59, 66–68). However, unlike the CDC guideline, these recommendations have drawn on a relatively narrow band of evidence. In particular, they have largely drawn on evidence about the actual consumption of opioids after surgery among American cohorts to determine how to manage a significant majority of patients' pain while limiting the number of unused opioids available for diversion (59, 67, 68). That is to say, these guidelines have been constructed with the primary aim of reducing the overall amount of opioids prescribed and consumed following surgery. While this is a worthwhile aim, it is unclear whether this should be the only—or the primary—aim of discharge prescribing guidelines. The total number or dosage of opioids prescribed to patients following surgery is, after all, an indirect proxy for patients' risk of opioid-related harm. Thus, future research evaluating the efficacy of interventions to reduce postoperative prescribing ought to directly measure rates of opioid-related harm alongside the total amount of opioids being prescribed. In addition to this, to ensure that prescribing practices do not over-correct, it is vital that such research is mindful of patients' pain and quality of life.

In addition, it is important to note that early guidelines have largely drawn conclusions about optimal prescribing practices by monitoring opioid consumption in the US—which is the epicenter of the global opioid crisis. While such an approach has shown the potential to reduce rates of excess prescribing, it is unclear that it is the most appropriate way to formulate recommendations for postoperative prescribing. More ambitious guidelines could anchor future recommendations to prescription practices or levels of consumption in nations that offer high quality surgical care while prescribing markedly fewer opioids after surgery. Countries like Sweden where prescriptions are filled at a seven-times lower rate than in North America, offer a vital source of evidence about how significantly opioid prescribing practices could be altered in countries like the US without compromising the quality of surgical care (55). Collecting data to inform the development of such prescribing recommendations is likely to require long-term international collaborations at a scale that has yet to emerge among researchers in this field. Such collaborations are necessary not only to inform the next wave of postoperative prescribing guidelines, but also to rigorously examine if implementing similarly restrictive prescribing practice is feasible across nations. To this end, the Consortium Against the overuse of Opioids in Surgery (CAOS) has been formed as a multinational (American, European, Oceania) initiative to address the research issues raised in this review.

## Author Contributions

All authors contributed to the conception, design of the study, and interpretation of the data. CS and LL were responsible for data and literature acquisition. CS wrote the first draft. All authors contributed to revising the manuscript for critically important intellectual content, read, and approved the submitted version.

### Conflict of Interest

LL reports personal fees from Arthro Therapeutics AB, GSK, Janssen Research & Development, Pfizer, and Regeneron, outside the submitted work. ES reports consulting fees unrelated to this work from Egalet, Inc. and the Mission Lisa Foundation. MD reports personal fees from Pfizer and grants from Medacta, outside the submitted work. PFMC reports personal fees from Stryker, Johnson & Johnson, and Kluwer, and grants from Medacta, outside the submitted work. The remaining authors declare that the research was conducted in the absence of any commercial or financial relationships that could be construed as a potential conflict of interest.

## References

[1] Berterame S Erthal J Thomas J Fellner S Vosse B Clare P. Use of and barriers to access to opioid analgesics: a worldwide, regional, and national study. Lancet. (2016) 387:1644–56. 10.1016/S0140-6736(16)00161-6 26852264

[2] Rudd RA Seth P David F Scholl L. Increases in drug and opioidinvolved overdose deaths—United States, 2010–2015. MMWR Morb Mortal Wkly. (2016) 65:1445–52. 10.15585/mmwr.mm655051e1 28033313

[3] Fischer BGJ Goldman B Kurdyak P Rehm J. Non-medical prescription opioid use, prescription opioid-related harms and public health in Canada: an update 5 years later. Can J Public Health. (2014) 105:e146–9. 10.17269/cjph.105.4143 24886852 PMC6972194

[4] Lalic S Ilomaki J Bell JS Korhonen MJ Gisev N. Prevalence and incidence of prescription opioid analgesic use in Australia. Br J Clin Pharmacol. (2019) 85:202–15. 10.1111/bcp.13792 30338545 PMC6303244

[5] Karanges EA Blanch B Buckley NA Pearson SA. Twenty-five years of prescription opioid use in Australia: a whole-of-population analysis using pharmaceutical claims. Br J Clin Pharmacol. (2016) 82:255–67. 10.1111/bcp.12937 26991673 PMC4917790

[6] Muller AE Clausen T Sjogren P Odsbu I Skurtveit S. Prescribed opioid analgesic use developments in three Nordic countries, 2006–2017. Scand J Pain. (2019) 19:345–53. 10.1515/sjpain-2018-0307 30677000

[7] Leifman HJ Drug-Related Deaths in Sweden–Estimations of Trends, Effects of Changes in Recording Practices and Studies of Drug Patterns. The Swedish Council for Information on Alcohol and Other Drugs (CAN): Stockholm (2016).

[8] National Safety Council Injury Facts. Avaiable online at: https://injuryfacts.nsc.org/all-injuries/preventable-death-overview/odds-of-dying/ (accessed October 7, 2019).

[9] Ray WA Chung CP Murray KT Hall K Stein CM. Prescription of long-acting opioids and mortality in patients with chronic noncancer pain. JAMA. (2016) 315:2415–23. 10.1001/jama.2016.7789 27299617 PMC5030814

[10] Bohnert ASB Ilgen MA. Understanding links among opioid use, overdose, and suicide. N Engl J Med. (2019) 380:71–9. 10.1056/NEJMra1802148 30601750

[11] The Council of Economic Advisers, Executive Office of the President of the United States The Underestimated Costs of the Opioid Crisis. (2017). Available online at: https://www.whitehouse.gov/sites/whitehouse.gov/files/images/TheUnderestimatedCostoftheOpioidCrisis.pdf (accessed October 7, 2019).

[12] Florence CS Zhou C Luo F Xu L. The economic burden of prescription opioid overdose, abuse, and dependence in the United States, 2013. Med Care. (2016) 54:901–6. 10.1097/MLR.0000000000000625 27623005 PMC5975355

[13] Levy B Paulozzi L Mack KA Jones CM. Trends in opioid analgesic-prescribing rates by specialty, U.S., 2007–2012. Am J Prev Med. (2015) 49:409–13. 10.1016/j.amepre.2015.02.020 25896191 PMC6034509

[14] Vowles KE McEntee ML Julnes PS Frohe T Ney JP van der Goes DNJP. Rates of opioid misuse, abuse, and addiction in chronic pain: a systematic review and data synthesis. Pain. (2015) 156:569–76. 10.1097/01.j.pain.0000460357.01998.f1 25785523

[15] World Health Organization The ICD-10 Classification of Mental and Behavioural Disorders: Clinical Descriptions and Diagnostic Guidelines. Geneva (1992).

[16] Kent ML Hurley RW Oderda GM Gordon DB Sun E Mythen M. American Society for enhanced recovery and perioperative quality initiative-4 joint consensus statement on persistent postoperative opioid use: definition, incidence, risk factors, and health care system initiatives. Anesth Analg. (2019) 129:543–52. 10.1213/ANE.0000000000003941 30897590 PMC6640123

[17] Dowell D Haegerich TM Chou R. CDC guideline for prescribing opioids for chronic pain–United States, 2016. JAMA. (2016) 315:1624–45. 10.1001/jama.2016.1464 26977696 PMC6390846

[18] Els C Kunyk D Lappi VG Sonnenberg B Hagtvedt R Sharma S. Adverse events associated with medium- and long-term use of opioids for chronic non-cancer pain: an overview of cochrane reviews. Cochrane Database Syst Rev. (2017) 10:CD012509. 10.1002/14651858.CD012509.pub2 29084357 PMC6485910

[19] Roughead EE Lim R Ramsay E Moffat AK Pratt NL. Persistence with opioids post discharge from hospitalisation for surgery in Australian adults: a retrospective cohort study. BMJ Open. (2019) 9:e023990. 10.1136/bmjopen-2018-023990 30992289 PMC6500207

[20] Sun EC Bateman BT Memtsoudis SG Neuman MD Mariano ER Baker LC. Lack of association between the use of nerve blockade and the risk of postoperative chronic opioid use among patients undergoing total knee arthroplasty: evidence from the marketscan database. Anesth Analg. (2017) 125:999–1007. 10.1213/ANE.0000000000001943 28430692

[21] Inacio MC Hansen C Pratt NL Graves SE Roughead EE. Risk factors for persistent and new chronic opioid use in patients undergoing total hip arthroplasty: a retrospective cohort study. BMJ Open. (2016) 6:e010664. 10.1136/bmjopen-2015-010664 27130165 PMC4853994

[22] Mohamadi A Chan JJ Lian J Wright CL Marin AM Rodriguez EK. Risk factors and pooled rate of prolonged opioid use following trauma or surgery: a systematic review and meta-(regression) analysis. J Bone Joint Surg Am. (2018) 100:1332–40. 10.2106/JBJS.17.01239 30063596

[23] Cron DC Englesbe MJ Bolton CJ Joseph MT Carrier KL Moser SE. Preoperative opioid use is independently associated with increased costs and worse outcomes after major abdominal surgery. Ann Surg. (2017) 265:695–701. 10.1097/SLA.0000000000001901 27429021

[24] Waljee JF Cron DC Steiger RM Zhong L Englesbe MJ Brummett CM. Effect of preoperative opioid exposure on healthcare utilization and expenditures following elective abdominal surgery. Ann Surg. (2017) 265:715–21. 10.1097/SLA.0000000000002117 28151795 PMC5767099

[25] Jain N Brock JL Phillips FM Weaver T Khan SN. Chronic preoperative opioid use is a risk factor for increased complications, resource use, and costs after cervical fusion. Spine J. (2018) 18:1989–98. 10.1016/j.spinee.2018.03.015 29709553

[26] Ben-Ari A Chansky H Rozet I. Preoperative opioid use is associated with early revision after total knee arthroplasty: a study of male patients treated in the veterans affairs system. J Bone Joint Surg Am Vol. (2017) 99:1–9. 10.2106/JBJS.16.00167 28060227

[27] Doan LV Wang J Padjen K Gover A Rashid J Osmani B. Preoperative long-acting opioid use is associated with increased length of stay and readmission rates after elective surgeries. Pain Med. (2019) 20:2539–51. 10.1093/pm/pny318 30802910

[28] Dasinger EA Graham LA Wahl TS Richman JS Baker SJ Hawn MT. Preoperative opioid use and postoperative pain associated with surgical readmissions. Am J Surg. (2019) 218:828–35. 10.1016/j.amjsurg.2019.02.033 30879796

[29] Lawrence JTR London N Bohlman HH Chin KR. Preoperative narcotic use as a predictor of clinical outcome: results following anterior cervical arthrodesis. Spine. (2008) 33:2074–8. 10.1097/BRS.0b013e3181809f07 18758363

[30] Kim Y Cortez AR Wima K Dhar VK Athota KP Schrager JJ. Impact of preoperative opioid use after emergency general surgery. J Gastrointest Surg. (2018) 22:1098–103. 10.1007/s11605-017-3665-x 29340924

[31] Cron DC Lee JS Dupree JM Syrjamaki JD Hu HM Palazzolo WC Provider characteristics associated with outpatient opioid prescribing after surgery. Ann Surg. 268:93–9. 10.1097/SLA.0000000000003013 PMC7055459 30247321

[32] Hilliard PE Waljee J Moser S Metz L Mathis M Goesling J. Prevalence of preoperative opioid use and characteristics associated with opioid use among patients presenting for surgery. JAMA Surg. (2018) 153:929–37. 10.1001/jamasurg.2018.2102 29998303 PMC6233789

[33] Menendez ME Ring D Bateman BT. Preoperative opioid misuse is associated with increased morbidity and mortality after elective orthopaedic surgery. Clin Orthop Relat Res. (2015) 473:2402–12. 10.1007/s11999-015-4173-5 25694266 PMC4457771

[34] Goplen CM Verbeek W Kang SH Jones CA Voaklander DC Churchill TA. Preoperative opioid use is associated with worse patient outcomes after total joint arthroplasty: a systematic review and meta-analysis. BMC Musculoskelet Disord. (2019) 20:234. 10.1186/s12891-019-2619-8 31103029 PMC6525974

[35] Bell KL Shohat N Goswami K Tan TL Kalbian I Parvizi J. Preoperative opioids increase the risk of periprosthetic joint infection after total joint arthroplasty. J Arthroplasty. (2018) 33:3246–51.e1. 10.1016/j.arth.2018.05.027 30054211

[36] Blevins Peratikos M Weeks HL Pisansky AJB Yong RJ Stringer EA Effect of preoperative opioid use on adverse outcomes, medical spending, and persistent opioid use following elective total joint arthroplasty in the United States: a large retrospective cohort study of administrative claims data. Pain Med. (2019) 23:pnz083 10.1093/pm/pnz083 PMC7060398 31120529

[37] Politzer CS Kildow BJ Goltz DE Green CL Bolognesi MP Seyler TM. Trends in opioid utilization before and after total knee arthroplasty. J Arthroplasty. (2018) 33:S147–53 e1. 10.1016/j.arth.2017.10.060 29198871

[38] Bannuru RR Osani M Vaysbrot E Arden N Bennell K Bierma-Zeinstra S. OARSI guidelines for the non-surgical management of knee, hip, and polyarticular osteoarthritis. Osteoarthritis Cartilage. (2019) 27:1578–89. 10.1016/j.joca.2019.06.011 31278997

[39] Smith SR Deshpande BR Collins JE Katz JN Losina E. Comparative pain reduction of oral non-steroidal anti-inflammatory drugs and opioids for knee osteoarthritis: systematic analytic review. Osteoarthritis Cartilage. (2016) 24:962–972. 10.1016/j.joca.2016.01.135 26844640 PMC4996269

[40] Krebs EE Gravely A Nugent S Jensen AC DeRonne B Goldsmith ES. Effect of opioid vs nonopioid medications on pain-related function in patients with chronic back pain or hip or knee osteoarthritis pain: the SPACE randomized clinical trial. JAMA. (2018) 319:872–82. 10.1001/jama.2018.0899 29509867 PMC5885909

[41] Hochberg MC Altman RD April KT Benkhalti M Guyatt G McGowan J. American college of rheumatology 2012 recommendations for the use of nonpharmacologic and pharmacologic therapies in osteoarthritis of the hand, hip, and knee. Arthritis Care Res. (2012) 64:465–74. 10.1002/acr.21596 22563589

[42] Smith SR Katz JN Collins JE Solomon DH Jordan JM Suter LG. Cost-effectiveness of tramadol and oxycodone in the treatment of knee osteoarthritis. Arthritis Care Res. (2017) 69:234–42. 10.1002/acr.22916 27111538 PMC5378156

[43] McAnally H. Rationale for and approach to preoperative opioid weaning: a preoperative optimization protocol. Perioper Med. (2017) 6:19. 10.1186/s13741-017-0079-y 29201359 PMC5700757

[44] Nguyen LC Sing DC Bozic KJ. Preoperative reduction of opioid use before total joint arthroplasty. J Arthroplasty. (2016) 31:282–7. 10.1016/j.arth.2016.01.068 27105557

[45] Sandhu H Underwood M Furlan AD Noyes J Eldabe S. What interventions are effective to taper opioids in patients with chronic pain? BMJ. (2018) 362:k2990. 10.1136/bmj.k2990 30262590

[46] Demidenko MI Dobscha SK Morasco BJ Meath THA Ilgen MA Lovejoy TI. Suicidal ideation and suicidal self-directed violence following clinician-initiated prescription opioid discontinuation among long-term opioid users. Gen Hosp Psychiatr. (2017) 47:29–35. 10.1016/j.genhosppsych.2017.04.011 28807135

[47] Compton WM Jones CM Baldwin GT. Relationship between nonmedical prescription-opioid use and heroin use. N Engl J Med. (2016) 374:154–63. 10.1056/NEJMra1508490 26760086 PMC11784537

[48] Eid AI DePesa C Nordestgaard AT Kongkaewpaisan N Lee JM Kongwibulwut M. Variation of opioid prescribing patterns among patients undergoing similar surgery on the same acute care surgery service of the same institution: time for standardization? Surgery. (2018) 164:926–30. 10.1016/j.surg.2018.05.047 30049481

[49] Hill MV McMahon ML Stucke RS Barth RJ Jr. Wide Variation and excessive dosage of opioid prescriptions for common general surgical procedures. Ann Surg. (2017) 265:709–14. 10.1097/SLA.0000000000001993 27631771

[50] Cartmill RS Yang DY Fernandes-Taylor S Kohler JE. National variation in opioid prescribing after pediatric umbilical hernia repair. Surgery. (2019) 165:838–42. 10.1016/j.surg.2018.10.029 30509750

[51] Mordecai L Reynolds C Donaldson LJ de CWAC. Patterns of regional variation of opioid prescribing in primary care in England: a retrospective observational study. Br J Gen Pract. (2018) 68:e225–33. 10.3399/bjgp18X695057 29440012 PMC5819988

[52] Australian Commission on Safety and Quality in Health Care The Third Australian Atlas of Healthcare Variation. (2018). Available online at: https://www.safetyandquality.gov.au/sites/default/files/migrated/The-Third-Australian-Atlas-of-Healthcare-Variation-2018.pdf (accessed October 7, 2019).

[53] Lindenhovius AL Helmerhorst GT Schnellen AC Vrahas M Ring D Kloen P. Differences in prescription of narcotic pain medication after operative treatment of hip and ankle fractures in the United States and The Netherlands. J Trauma. (2009) 67:160–4. 10.1097/TA.0b013e31818c12ee 19590328

[54] Li RJ Loyo Li M Leon E Ng CWK Shindo M Manzione K. Comparison of opioid utilization patterns after major head and neck procedures between hong kong and the United States. JAMA Otolaryngol Head Neck Surg. (2018) 144:1060–5. 10.1001/jamaoto.2018.1787 30193293 PMC6248185

[55] Ladha KS Neuman MD Broms G Bethell J Bateman BT Wijeysundera DN. Opioid prescribing after surgery in the United States, Canada, and Sweden. JAMA Netw Open. (2019) 2:e1910734. 10.1001/jamanetworkopen.2019.10734 31483475 PMC6727684

[56] Bicket MC Long JJ Pronovost PJ Alexander GC Wu CL. Prescription opioid analgesics commonly unused after surgery: a systematic review. JAMA Surg. (2017) 152:1066–71. 10.1001/jamasurg.2017.0831 28768328 PMC5701659

[57] Feinberg AE Chesney TR Srikandarajah S Acuna SA McLeod RS Best practice in surgery G. opioid use after discharge in postoperative patients: a systematic review. Ann Surg. (2018) 267:1056–62. 10.1097/SLA.0000000000002591 29215370

[58] Sabatino MJ Kunkel ST Ramkumar DB Keeney BJ Jevsevar DS. Excess opioid medication and variation in prescribing patterns following common orthopaedic procedures. J Bone Joint Surg Am. (2018) 100:180–8. 10.2106/JBJS.17.00672 29406338 PMC6818977

[59] Thiels CA Ubl DS Yost KJ Dowdy SC Mabry TM Gazelka HM. Results of a prospective, multicenter initiative aimed at developing opioid-prescribing guidelines after surgery. Ann Surg. (2018) 268:457–68. 10.1097/SLA.0000000000002919 30004924

[60] Vu JV Howard RA Gunaseelan V Brummett CM Waljee JF Englesbe MJ. Statewide implementation of postoperative opioid prescribing guidelines. N Engl J Med. (2019) 381:680–2. 10.1056/NEJMc1905045 31412184 PMC7160762

[61] Khan NF Bateman BT Landon JE Gagne JJ Association of opioid overdose with opioid prescriptions to family members. JAMA Intern Med. (2019) 179:1186–92. 10.1001/jamainternmed.2019.1064 PMC6593630 31233088

[62] Chou R Turner JA Devine EB Hansen RN Sullivan SD Blazina I. The effectiveness and risks of long-term opioid therapy for chronic pain: a systematic review for a national institutes of health pathways to prevention workshop. Ann Intern Med. (2015) 162:276–86. 10.7326/M14-2559 25581257

[63] Wick EC Grant MC Wu CL. Postoperative multimodal analgesia pain management with nonopioid analgesics and techniques: a review. JAMA Surg. (2017) 152:691–7. 10.1001/jamasurg.2017.0898 28564673

[64] Wu CL King AB Geiger TM Grant MC Grocott MP Gupta R. American society for enhanced recovery and perioperative quality initiative joint consensus statement on perioperative opioid minimization in opioid-naïve patients. Anesth Analg. (2019) 129:567–77. 10.1213/ANE.0000000000004194 31082966 PMC7261519

[65] Bohnert ASB Guy GP Jr Losby JL. Opioid prescribing in the United States before and after the centers for disease control and prevention's 2016 opioid guideline. Ann Intern Med. (2018) 169:367–75. 10.7326/M18-1243 30167651 PMC6176709

[66] Overton HN Hanna MN Bruhn WE Hutfless S Bicket MC Makary MA. Opioid-prescribing guidelines for common surgical procedures: an expert panel consensus. J Am Coll Surg. (2018) 227:411–8. 10.1016/j.jamcollsurg.2018.07.659 30118896 PMC6353661

[67] Howard R Waljee J Brummett C Englesbe M Lee J. Reduction in opioid prescribing through evidence-based prescribing guidelines. JAMA Surg. (2018) 153:285–7. 10.1001/jamasurg.2017.4436 29214318 PMC5885924

[68] Hill MV Stucke RS Billmeier SE Kelly JL Barth RJ Jr. Guideline for discharge opioid prescriptions after inpatient general surgical procedures. J Am Coll Surg. (2018) 226:996–1003. 10.1016/j.jamcollsurg.2017.10.012 29198638

